# Tennessee Dental Association

**Published:** 1903-12

**Authors:** A. Sydney Page

**Affiliations:** Secretary


### TENNESSEE DENTAL ASSOCIATION.

The thirty-sixth annual meeting of the Tennessee Dental Association was held at Lookout Inn, Chattanooga, Tenn., July 23 to 25, 1903, Dr. W. K. Slater, of Knoxville, presiding. The meeting was very satisfactory, pronounced by many to be the most interesting held for years. The attendance was good, including quite a number of the profession from other States. Some who would have otherwise attended were kept away on account of the National meetings at Asheville being held at the same time.

Ten excellent papers were read and thoroughly discussed, the President's Address being an especially happy effort, his principal theme being "Dental Ethics."

The clinics were presided over by Dr. A. A. McLanahan, of Springfield, and of course that part of the programme was not a failure by any means, many very interesting clinics being given. The "inlay man" was very much in evidence.

Treasurer Dr. J. D. Towner, of Memphis, reported the finances of the Association in good condition, with $156.52 on hand.

Twelve new members were added to the roll, most of them being young men, which is, we take it, a good omen of better things ahead.

The Association sent a full delegation to the National and Southern Branch meetings at Asheville.

The following officers were elected for the ensuing year:

President, Dr. R. Boyd Bogle, Nashville, Tenn.; First Vice-President, Dr. J. D. Towner, Memphis, Tenn.; Second Vice-President, Dr. A. J. Cottrell, Knoxville, Tenn.; Recording Secretary, Dr. J. T. Crews, Jackson, Tenn.; Corresponding Secretary, Dr. W. K. Slater, Knoxville, Tenn.; Treasurer, Dr. W. P. Sims, Nashville, Tenn.; Member of Executive Committee, Dr. P. M. Joyner, Union City, Tenn.; Chairman Clinic Committee, Dr. A. A. McLanahan, Springfield, Tenn.

*Essay Committee.*—Dr. W. C. Gillespie, Nashville, Tenn.; Dr. A. J. Cottrell, Knoxville, Tenn.; Dr. J. D. Towner, Memphis, Tenn.

The next meeting will be held at Jackson, Tenn., on a date to be selected by the Executive Committee.

A. Sydney Page,
*Secretary.*

